# The prevalence of polycystic ovary syndrome in reproductive-aged women of different ethnicity: a systematic review and meta-analysis

**DOI:** 10.18632/oncotarget.19180

**Published:** 2017-07-12

**Authors:** Tao Ding, Paul J. Hardiman, Irene Petersen, Fang-Fang Wang, Fan Qu, Gianluca Baio

**Affiliations:** ^1^ Department of Statistical Science, University College London, London, United Kingdom; ^2^ Institute for Women's Health, University College London Medical School, London, United Kingdom; ^3^ Department of Primary Care and Population Health, University College London, London, United Kingdom; ^4^ Women's Hospital, School of Medicine, Zhejiang University, Hangzhou, China

**Keywords:** polycystic ovary syndrome, prevalence, ethnicity, systematic review

## Abstract

The prevalence of PCOS was investigated in many studies in different continents. However, there is no established prevalence of PCOS for distinct ethnic groups. In the current analysis, we conducted searches in PubMed, The Cochrane Library, EMBASE, CINAHL up to Jan. 2017 to identify studies reporting prevalence of PCOS in the general female population. Forty-two studies were identified, with 13 eligible for evidence synthesis. The prevalence among different ethnicity was estimated using random effect modelling. Our results suggested the lowest prevalence in Chinese women(2003 Rotterdam criterion: 5.6% 95% interval: 4.4–7.3%), and then in an ascending order for Caucasians (1990 NIH criterion: 5.5% 95% interval: 4.8–6.3%), Middle Eastern (1990 NIH 6.1% 95% interval: 5.3–7.1%; 2003 Rotterdam 16.0% 95% interval: 13.8–18.6%; 2006 AES 12.6% 95% interval: 11.3–14.2%), and Black women (1990 NIH: 6.1% 95% interval: 5.3–7.1%).There is variation in prevalence of PCOS under different diagnostic criteria and across ethnic groups. This emphasises the need for ethnicity-specific guidelines for PCOS to prevent under- or over-diagnosis of the condition given that under-diagnosis may lead to rapid conversion of metabolic disorders for patients whereas over-diagnosis may exert negative psychological effects on patients which worsens the major symptoms of PCOS.

## INTRODUCTION

Polycystic ovary syndrome (PCOS) is a heterogeneous endocrine disorder, leading to several health complications, including menstrual dysfunction, infertility, hirsutism, acne, obesity, and metabolic syndrome [1]. However, its pathophysiology remains largely unknown but many believe that PCOS appears to be familial, with its various aspects differentially inherited from one generation to the next [2]. Although more than 100 candidate genes have been investigated, and the potential for gene discovery to improve diagnosis and treatment of PCOS is promising, there is much to be done before the current findings can be applied in clinical practice [3, 4]. The three major diagnostic criteria of PCOS widely followed are criteria raised by National Institutes of Health(NIH) [5], 2003 Rotterdam Consensus raised by European Society of Human Reproduction and Embryology (ESHRE) and American Society for Reproductive Medicine (ASRM) [6, 7] and criteria raised by Androgen Excess Society (AES) [8].

It is known that based particularly on ancestry and geographic segregation, the world's populations vary in physical, behavioural and social distinctiveness due to natural selection of genes and adaptation to environmental conditions, which then influences disease phenotype. There is emerging evidence that ethnicity is closely associated with PCOS phenotype due to different genetic and environmental propensity to metabolic and hormonal aberrations [9–11]. As early as in 1992, it was found that obesity and hirsutism are associated with some genetic factors [12], and consequently, the ethnic background of women with PCOS needs to be considered in studies that investigate the metabolic parameters [13]. A new study has suggested that Hispanic women with PCOS generally present the most severe phenotype both in terms of hyperandrogenism and metabolic features whereas non-Hispanic Black women demonstrate an overall milder clinical presentation of PCOS than Hispanics and non-Hispanic White women with respect to some aspects [14].

Ethnicity-specific guidelines of PCOS are potentially in need to identify anthropometric thresholds and phenotypic expression for better screening and diagnosis in high-risk ethnic groups [15, 16]. However, to our knowledge, the prevalence of PCOS in distinct ethnic groups has not been established. To address this issue, we therefore, performed a comprehensive literature review to collect relevant studies and establish the prevalence of PCOS in different ethnic groups by using suitable statistical modelling.

## RESULTS

### Eligible articles

**Figure 1:**
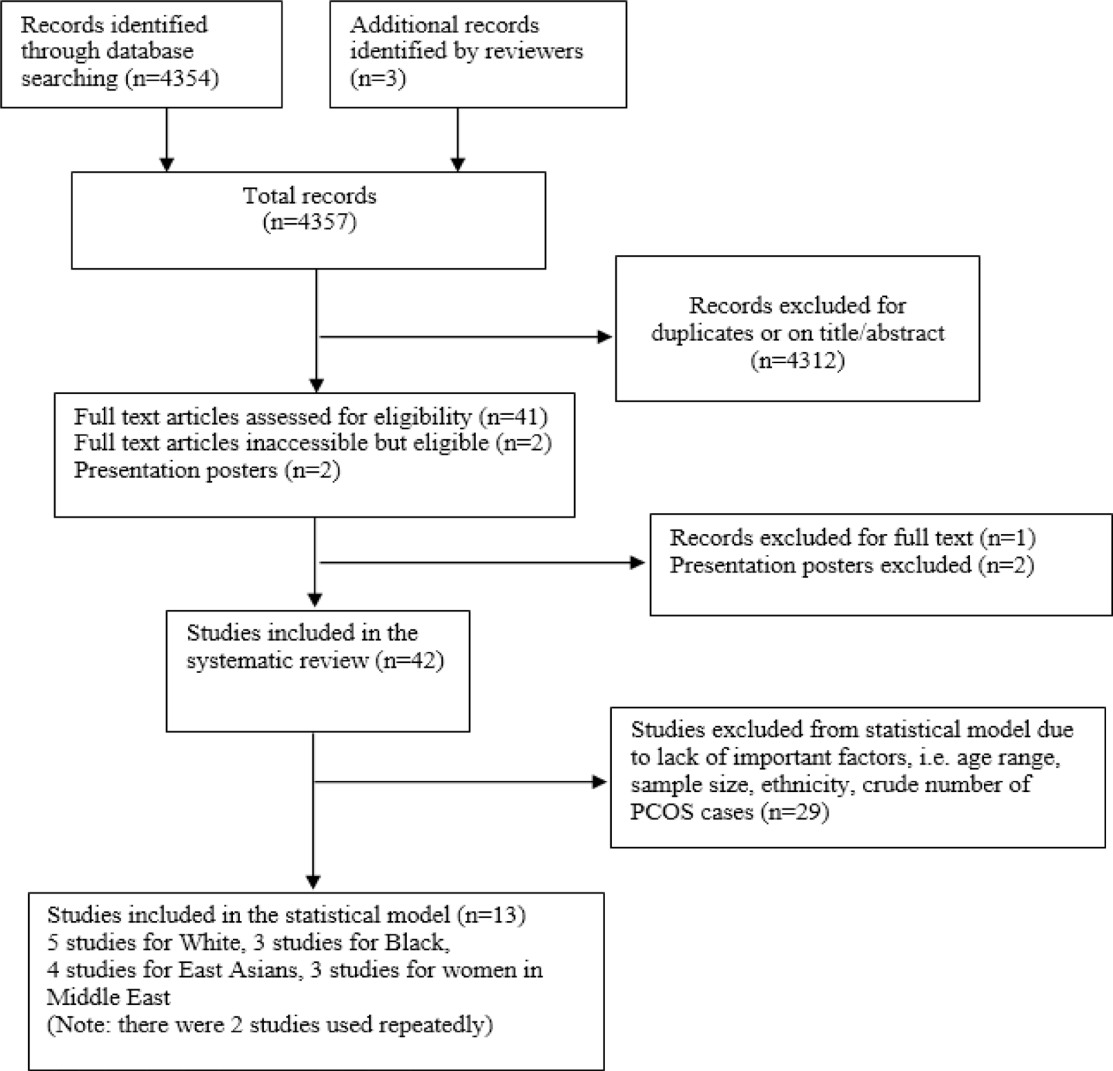
Flow chart for systematic review and statistical model

Our search initially produced 4354 citations in total, and 45 studies were identified. We excluded 2 presentation posters as these studies did not appear to have been published in peer reviewed journals. One study was also excluded after full-text screening because it evaluated prevalence of PCOS in patients with type 2 diabetes mellitus. Figure 1 presents a consort diagram summarising our search.

### Characteristics of prevalence studies

We found 42 studies (see Supplementary Table 1) evaluating the prevalence of PCOS but we only retrieved the full text for 40 of them, with the remaining 2 articles inaccessible [17, 18]: there were no links to follow on the PubMed for these two articles. However, in the abstracts of these articles, Jiao *et al*. [17] clearly presented source of recruitment, sample size, age range, ethnicity, definition of PCOS, prevalence data and the corresponding crude number of PCOS cases; Sung *et al*. [18] provided source of recruitment, age range, definition of PCOS, prevalence data, with the crude number of PCOS cases and sample size irretrievable.

Geographically speaking, there were 10 studies in Americas, 7 in Europe, 11 in Asia, 10 in Middle East and 4 in Oceania. The sample size ranged from 50 to 12171830.

### Prevalence data for different ethnicity

Studies scoring 5 and above were evaluated to examine the eligibility of inclusion in our model (*n* = 27). Studies that did not fit the general pattern as the majority of other studies in the same category were further excluded. For example, Lauritsen *et al*. [19] was removed due to the fact that all the other four studies in the Europe used the 1990 NIH while it followed the 2003 Rotterdam. Hashemipour *et al*., [20], Mehrabian *et al*., [21], Asgharnia *et al*., [22] and Musmar *et al*., [23] were excluded because the age range of the sample population was much narrower (i.e. 17–18yrs) compared with the rest studies in Middle East region. Zhuang *et al*. [24]was also removed for using a wider than reproductive age range (12–44 years), with the prevalence of PCOS peaking at the age band of 15–19 years. Statistical modelling was not performed for South Asians (India and Sri Lanka) and Australians because all studies in the same region used different age ranges (i.e. 15–18 yrs, 18–24 yrs). As a result, in terms of age, all the studies considered for our statistical modelling included study population with an age range of either 17–45, 18–45, 19–45 years or a median between 27 and 33, with an inter-quartile range of 9–13 which can be approximated to reproductive age range (see studies with an asterisk in Supplementary Table 1). It should also be noted that we did not model prevalence for certain ethnic groups (i.e. Mexicans, Thai) as there was only one study available. The reason for this is that if the result in a single study is spontaneous and unreliable we have no choice but to use it as our “best estimate”, which is undoubtedly questionable. It was also spotted that the prevalence and the crude number of PCOS cases do not match under the 2006 AES criteria described by Mehrabian *et al*. [21].

After further investigation of features of each available study, we noticed that some studies used a single ethnic group while others used a mixture of different ethnic groups. For example, two US prevalence study [25, 26] reported using both Black and White, while the study conducted in Spain used Caucasian females only [27].

### Main outcomes from statistical modelling

The results of estimated prevalence of PCOS in general female population obtained from our model were shown in the Table 1.

**Table 1:** Estimated prevalence of PCOS in unselected female population

Ethnicity	Estimated prevalence (%) of PCOS in general female population (with 95% CrI)		
	1990 NIH	2003 Rotterdam	2006 AES
White (Caucasian)	5.5 (4.8–6.3)	−	−
Black (African-American and Afro-Brazilian)	7.4 (6.3–8.7)	−	−
Chinese	−	5.6 (4.4–7.3)	−
Middle East (Iranian and Turkish)	6.1 (5.3–7.1)	16.0 (13.8–18.6)	12.6 (11.3–14.2)

For the 1990 NIH, the prevalence of PCOS for White women was estimated at 5.5% (95% CrI: 4.8–6.3%). The corresponding figures for Black women and women residing in the Middle East are 7.4% (95% CrI: 6.3–8.7%) and 6.1% (95% CrI: 5.3–7.1%), respectively.

Using the 2003 Rotterdam, prevalence estimation is only feasible for Chinese women (5.6%, 95% CrI: 4.4–7.3%) and women in the Middle East (16.0%, 95% CrI: 13.8–18.6%). The prevalence of PCOS in the Middle East women almost triples that in female Chinese.

As most studies in Middle East provide prevalence data using all the three major diagnostic criteria of PCOS, we were able to extrapolate prevalence for females in Middle East according to different criteria. The prevalence of PCOS is 6.1% (95% CrI: 5.3–7.1%) under the 1990 NIH, 16.0% (95% CrI: 13.8–18.6%) under the 2003 Rotterdam, and 12.0% (95% CrI: 11.3–14.2%) under the 2006 AES for females in Middle East. The prevalence under Rotterdam more than doubles that under the 1990 NIH, with the prevalence under the 2006 AES lying in-between.

## DISCUSSION

The current study suggested that the prevalence of PCOS reported in the database studies are generally lower compared with that reported in the community studies, underlying the fact that PCOS is a syndrome without much public awareness and PCOS patients often do not seek care. Moreover, for the same ethnicity, the prevalence of PCOS was estimated to be the highest for the 2003 Rotterdam and the lowest for the 1990 NIH. This confirms the results in another study where prevalence studies were categorised by geographical locations [28]. While Bozdag et al. [28] estimated continental-specific prevalence, it is common that nowadays individuals with different ethnic backgrounds reside in the same region due to globalisation. Therefore, our current study is advantageous in terms of providing ethnicity-specific estimates.

We found that Caucasian females living in the US and Europe are less likely to develop PCOS compared with females residing in the Middle East whereas Black women (the majority are African-Americans and Afro-Brazilians) tend to have the highest risks of developing PCOS. The upper bound of the 95% CrI of the PCOS prevalence for White females is the same as the lower bound of that for Black females, suggesting that White and Black females have substantially different risks of developing PCOS. For Chinese women, even using the 2003 Rotterdam, the prevalence is merely 5.6%, which is comparable with the prevalence for White females under the 1990 NIH. The 2003 Rotterdam has the broadest spectrum, so not surprisingly, it is expected that the prevalence under the 2006 AES and the 1990 NIH would be even smaller for Chinese females, had data been available. Therefore, Chinese women were suggested to be at a lower risk of PCOS compared with other ethnic groups. It should be noted that for females residing in the Middle East, the credible intervals of the prevalence estimates under the 1990 NIH and the 2006 AES do not overlap, indicating that the prevalence of PCOS for this ethnicity is notably different according to these two definitions.

In general, we would expect that under the same diagnostic criterion of PCOS, Chinese women are at a lowest risk of developing PCOS, and then in an ascending order through Caucasian women and females residing in the Middle East, with Black women having the highest risks of developing this syndrome.

The genetic ancestry data may be used to interpret the phenotypic variability associated with PCOS to a greater extent than self-reported ethnicity [29]. There are evidences for genetic influence based on European ethnicity in women with PCOS and a genetic component in the phenotypic features of PCOS within a mixed European population [30]. It was found that the risk variants associated with PCOS in Korean women were not replicated in women of European ethnicity [31]. In North India, clear different phenotypes of PCOS were emerging, probably due to ethnic variants [32]. The differences in phenotype and clinical symptoms of PCOS related to the clinical, hormonal, and metabolic characteristics among various ethnic backgrounds, including Hispanics, African Americans, Asians, and Indians, need to be considered when assessing and treating these individuals [33]. Particularly, women of different ethnicities had different presentations of clinical hyperandrogenism such as hirsutism [34], which strongly suggests that clinical hyperandrogenism related history taking and physical examination should vary from patient to patient according to different ethnicities. Taken together, the implications of ethnic variation on screening and diagnosis, management priorities and response to treatment should be taken into account when managing women from distinct ethnic backgrounds, as well as in developing management guidelines of PCOS.

It is also worth mentioning that even for the same ethnic group (i.e. women in the Middle East), there is huge variation in the prevalence of PCOS based on different diagnostic criteria. This potentially indicated the issue of under- or over-diagnosis of this condition at present. Given that the major concern for women with PCOS is the long-term metabolic risk, the clinical management of PCOS is suggested to be at the earliest possible when a diagnosis is confirmed in order to prevent the rapid conversion into complications such as type 2 diabetes [35]. However, on the contrary, healthcare workers should also be aware that the over-diagnosis of PCOS may exert negative psychological impact on women with symptoms indicative of PCOS. Psychological disorders may worsen some major symptoms of PCOS (i.e. menstrual dysfunction) and increase the chance for a potential case to be qualified as a true case. This is supported by a recent study stating that high stress is significantly associated with occurrence of severe dysmenorrhoea and irregular cycles [36].

The strict quality assessment resulted in only a limited number of eligible studies for inclusion in the statistical model, with a relatively small sample size reported by each study (e.g. between 154 and 15924). It is suggested that if the effect across studies is consistent, we would consider that the summary estimate is robust whereas in contrast, if there are substantial differences across studies, we may need to account for the dispersion. However, the problem arises because when there are few studies to work with, we may not know how the actual dispersion of studies look like. Therefore, we attempted to use Bayesian methods to address this issue. The potential drawback of this method is that the extrapolation of prevalence was largely driven by the prior distributions, which complemented the data. Nonetheless, although the prior distributions included may have some influence on the pooled estimates, we referred to experts’ opinions to inform our model parameters. For example, the prevalence of PCOS in the general population is expected to be within a reasonable range of 2–20% and this information was represented by some suitable prior distributions (i.e. bound the prevalence within this range with some variability). A notable advantage of using Bayesian approach is that with the application of simulation, the prior distributions can be updated by the observed data to generate some posterior distributions of the parameters of interest (in our case, prevalence of PCOS in different ethnicity). As a consequence, random sampling with a large sample size drawn from the posterior distributions was feasible, potentially providing more sensible results. Moreover, in our analysis, different versions of priors were attempted and pooled estimates were obtained from model averaging. Models with smaller Deviance Information Criterion (DIC), which indicates a better fit, were weighted up while models with larger DIC were weighted down. In this way, we did not discard information from models which have slightly higher DIC but give reasonable prevalence estimates and 95% credible interval. This improved the accuracy of our estimation.

As there are few instruments specifically designed (i.e. Newcastle-Ottawa scale for case-control and cohort studies) for prevalence studies, the formal evaluation of included studies was challenged. For example, few studies reported using sample size calculation and random sampling scheme. It was suggested by Munn. Z *et al*., [37] that the followings are all essential factors to be considered for prevalence studies: sampling scheme, sample representativeness, recruitment strategy, sample size calculation, description of study subjects and settings, response rate, standard criteria used for measurement of a specific condition, reliable measurement instrument, appropriate statistical analysis. However, few studies met all of the above criteria.

Although there are some inherent challenge, the results from the current analysis have suggested that using the same diagnostic standard, Chinese women would have the lowest risks of developing PCOS, and then in an ascending order by, Caucasian women and women residing in the Middle East, with Black women having the highest risk of developing this syndrome. Considering the wide variation in the clinical presentations associated with PCOS among distinct ethnicity, there is an urgent need for the establishment of ethnicity-specific guidelines for this condition. This may help to prevent the under- or over-diagnosis of PCOS. Further research into the community prevalence of PCOS in different ethnic populations may need to be warranted to provide sufficient data for prevalence extrapolation.

## MATERIALS AND METHODS

### Search strategy and eligibility criteria

A literature review for prevalence studies of PCOS was conducted in the following electronic databases: PubMed, The Cochrane Library, EMBASE, and CINAHL. These are common databases used for searching medical literature. The following combinations of essential search words were used to identify studies evaluating prevalence of PCOS:

### ((Stein-Leventhal syndrome) OR (polycystic ovary syndrome)) AND ((prevalence) OR (incidence) OR (epidemiology))

The search was restricted to English language only. Only studies that assessed the prevalence of PCOS in unselected general female population (excluding studies which included patients seeking medical care services for particular diseases, i.e. patients with type 2 diabetes mellitus) up to January 2017 were included. Papers with irrelevant titles or abstracts, for example, reviews of epidemiology of PCOS or, prevalence studies of PCOS-related diseases, were excluded. The reference lists of included studies and relevant systematic reviews were searched in order to locate other potential eligible articles. Three reviewers (T.D., F.W. and G.B.) independently screened and selected the articles and disagreement was resolved by consensus with P.J.H. and F.Q.

### Quality assessment

To ensure that the studies included in our statistical model provided quality data, we performed a methodological evaluation for the studies. As there are few systematic assessment criteria (i.e. Newcastle-Ottawa scale for case-control and cohort study) specifically designed for prevalence studies, we referred to both the Newcastle-Ottawa scale and the Joanna Briggs Institute prevalence critical appraisal [37] tools but modified some of the items within each category for methodological evaluation. Two independent reviewers (T.D. and G.B.) appraised all the articles. Inter-reviewer agreement of 0.95 was reached and disagreement was resolved by consensus (T.D., F.W., G.B., P.J.H. and F.Q.). See Supplementary Table 2 for more details of our quality assessment.

We considered studies that had a total score of below 5 as poor quality ones and therefore excluded them from our statistical model. They were removed because the following important factors were not clearly stated: (i) age range; (ii) sample size (for studies using multiple ethnic groups, sample size for distinct ethnic group needs to be reported; (iii) diagnostic standards do not follow three major criteria of PCOS; (iv) ethnicity.

### Data extraction

The following information was extracted from each available study:(i) General characteristics of the study (author, publication year, study period and location); (ii) Characteristics of sample population (recruitment and sampling methods, sample size, number of PCOS cases, age range and ethnicity of sample population); (iii) Definition of PCOS (1990 NIH, Rotterdam, AES, ICD-9 codes, medical diagnosed PCOS, clinical PCOS, self-reported PCOS). Data extraction was performed by T.D., and G.B. independently. Discrepancies were resolved by consensus (T.D., F.W., G.B., P.J.H. and F.Q.).

Studies were categorised according to geographical locations, which may potentially distinguish different ethnic groups, as it seems reasonable to assume that there is ethnic variation in the prevalence of PCOS [15].

We referred to the classification of ethnic category by National Health Service in the UK [38] and categorised studies strictly by ethnicity. All studies providing prevalence data for the same ethnicity were classified into the same category regardless of country origin. For studies where sample population consisted of more than one ethnicity (i.e. both Black and White), we used them repeatedly and extracted raw number of PCOS cases and corresponding sample sizes for different ethnicity separately. The raw numbers of PCOS in the sample of each study were extracted to be used for our model. For studies where the number of PCOS cases is not provided and the prevalence of PCOS is based on simulations, the raw number of PCOS cases was calculated and rounded to the nearest integer. Studies with an asterisk in the Supplementary Table 1 were included in our model.

### Data analysis

We analysed the data using a Bayesian hierarchical model. In a nutshell, the Bayesian approach to statistical inference [39–41] is based on the premise that both *sampling variability* (i.e. due to individual variations in the observed data) and *epistemic uncertainty* (i.e. due to our imperfect knowledge of model parameters, such as population prevalence) are modelled using probability distributions. These are used to describe the state of science currently available. Before observing the data, the modellers specify a ‘prior’ distribution for the quantity of interest, typically unobservable population parameters, to represent the current uncertainty. This prior is updated into a ‘posterior’ distribution through the application of Bayesian theorem, after the data are observed. The formal inclusion of prior information can be beneficial particularly in cases where the observed evidence is limited and non-conclusive. Practically, Bayesian analysis is typically performed using a simulation approach in which samples from the posterior distributions are obtained using computer algorithms. These can be used to quantify the updated level of uncertainty in the parameters of interest, e.g. by computing relevant summaries such as the mean or the interval containing most (e.g. 95%) of the posterior distribution.

We use a ‘hierarchical’ Bayesian model with a two-fold objective: firstly, we want to complement the limited information obtained by the literature review (see Results section) by explicitly modelling the prevalence parameters so as to reflect the assumption that the prevalence of PCOS is within a reasonable range in the general population, regardless of ethnicity. Secondly, the inclusion of ‘random effects’ (which renders our model ‘hierarchical’) allow us to account for correlation induced by clustering, e.g. within geographical areas or common background characteristics.

In our analysis, the prevalence estimates were obtained using the mean and the 2.5^th^ and 97.5^th^ percentiles of the posterior distribution (95% credible interval, CrI); these can be considered as a Bayesian counterpart to the lower and upper bound of the traditional 95% confidence interval. We attempted different versions of priors for our model to assess the robustness of the results. This includes normal distribution prior for ethnicity-specific prevalence at population level. The nature of the normal distribution allows certain degree of variability around a centred mean and we considered different mean prevalence within a reasonable range of 2–20% (A normal distribution with a mean of 0.1 and a variance of 0.0001 represents that the population level mean prevalence is 10% with a standard deviation from the mean of 0.01 (i.e. 95% CI: 8–12%)). The prior for the population-level prevalence is complemented by uniform (A Uniform distribution assumes that all values within a range are equally likely) or half-Cauchy (A half-Cauchy distribution is characterised by heavy tails (compared to a normal distribution) to allow for outliers and accommodating small variances close to zero (in our case, the variance for the population level mean prevalence is considered to be small as the mean prevalence itself is not large)) prior distribution for the population-level variance. The different specifications of our model (upon varying the prior distributions) were assessed using the DIC [42]– this is a measure of model fitting, with lower values indicating a better fit. However, because of the limited amount of information, rather than simply selecting one model (which could well be the least worse in a set of not entirely satisfactory models), we combined the possible formulations by weighting them using a function of their computed DIC. This produced a ‘model average’ describing our best assessment, given the limited evidence observed and a set of reasonable prior assumptions. Detailed explanations for our Bayesian hierarchical model and prior specifications are provided in Supplementary Figure 1.

The analyses were performed using simulation methods in JAGS, a specialised software used to obtain simulations from arbitrarily complex models, interfaced with R version 3.3.2 (The R Foundation for Statistical Computing). Two Markov chains ran simultaneously with different initial values selected arbitrarily for convergence purpose. A total of 40,000 simulations per chain were generated and the first 10,000 in the burn-in period were discarded.

## SUPPLEMENTARY MATERIALS FIGURES AND TABLES







## Abbreviations

PCOS: Polycystic ovary syndrome
NIH: National Institutes of Health
ESHRE: 2003 Rotterdam Consensus raised by European Society of Human Reproduction and Embryology
ASRM: American Society for Reproductive Medicine
AES: criteria raised by Androgen Excess Society
DIC: Deviance Information Criterion
