# Supplementary material for: The prevalence of polycystic ovary syndrome in reproductive-aged women of different ethnicity: a systematic review and meta-analysis
Source: Oncotarget. 2017 Jul 12;8(56):96351–8. doi: 10.18632/oncotarget.19180 (PMC5707105; doi:10.18632/oncotarget.19180)
Supplement: Supplementary file 1 [file oncotarget-08-96351-s001.pdf]

The prevalence of polycystic ovary syndrome in reproductive-aged women of different ethnicity: a systematic review and meta-analysis

SUPPLEMENTARY MATERIALS

The model includes several modules. Suppose there are I studies in total for the White, and for each studies $i = 1,...,I$ we observed the number of PCOS cases out of the total unselected female population. A Binomial distribution was used to model these studies:

$$x_i \sim Binomial(\beta_i, m_i) \quad (1)$$

β_i represents the probability of developing PCOS for White in study. i. Because the outcome is binary, which means that individuals can either be a PCOS case or not, we used logistic regression to model the pooled mean probability of developing PCOS for the White population (study-specific probability). We further assumed that random variables obtained after logit scale transformation follows a normal distribution:

$$logit(\beta_i) = \gamma_i \ Normal(\mu_\gamma, \sigma_\gamma^2) \quad (2)$$

μ_γ represents the pooled mean probability of developing PCOS on the logit scale for White population. In order to calculate the probability of PCOS, we need to rescale it to estimate:

$$p = \frac{\exp(\mu_\gamma)}{1+\exp(\mu_\gamma)} \quad (3)$$

where p is the pooled mean probability of PCOS for White population as a whole

This module is completed by including some reasonable prior distributions to μ_γ and σ_γ Markov Chain Monte Carlo methods were then performed in JAGS (interfaced with R) so the prior distributions can be updated by the observed data to generate some posterior distributions from which random samples of parameters of interest (i.e. prevalence of PCOS) can be drawn.

We tested several versions of prior distributions for this model by attempting a range of values for p and k in the following formula:

$$\mu_\gamma \sim Normal(p, \sigma_\gamma^2) \quad (4)$$

$$\sigma_\gamma \sim Uniform(0, k) \quad (5)$$

The prior distribution we included are based on some reasonable subjective belief. Given thatwe do not expect very high risk of developing PCOS for women, i.e. >20% in the general population. The values of were chosen to be within a reasonable range, i.e. 2-20% and different values were tested in a descending order (from largest value to smallest value). For example, we may start from 15% and go down to 12%, 9%, 6%, 3% to see which values of p produce better model fit statistics. It should be noted that μ_γ and σ_γ are on a logit scale, so even k= 2 represents a large variance.

However, as previously, we assumed that σ_γ follows a uniform distribution, it potentially indicates a problem that the simulation processes tend to be largely influenced by k where k is the upper bound of the uniform distribution, i.e.*Uniform* (0, k) Therefore, we decided to attempt half-Cauchy distribution for :

$$\mu_\gamma \sim Normal(p, \sigma_\gamma^2) \quad (6)$$

$$\sigma_\gamma = \frac{|Z_r|}{\sqrt{\varepsilon_r}} \quad (7)$$

$$Z_r \sim Normal(0, \sigma_{z_r}^2) \quad (8)$$

$$\varepsilon_r \sim Gamma(0.5, 0.5) \quad (9)$$

$$\sigma_{z_r}^2 = \frac{1}{B_r^2} \quad B_r \sim Uniform(0, 0.5) \quad (10)$$

The half-Cauchy distribution is advantageous in terms of allowing for outliers and accommodating small variances close to zero.

The statistical software provided deviance information criterion (DIC) of each model, which is a measurement of the goodness of fit of the model to the data, with lower values indicating a better fit. We attempted different versions of priors and then integrated results from various models by model averaging. Models with smaller DIC were weighted up while models with larger DIC were weighted down. The following formula was used to compute the weight of each model we included after statistical reasoning:

$$w_h = \frac{exp(-0.5\Delta DIC_h)}{\sum_{h=1}^{H} exp(-0.5\Delta DIC_h)} \quad (11)$$

where $\Delta DIC_h = min_h(DIC_h) - DIC_h$ and $h = 1,...H$, indicating the set of models

The same statistical principles were applied to obtain estimates of prevalence for other ethnic groups.

SUPPLEMENTARY REFERENCES

1. Knochenhauer ES, Key TJ, Kahsar-Miller M, Waggoner W, Boots LR, Azziz R. Prevalence of the polycystic ovary syndrome in unselected black and white women of the southeastern United States: a prospective study. The Journal of clinical endocrinology and metabolism. 1998; 83: 3078–3082.
2. Azziz R, Woods KS, Reyna R, Key TJ, Knochenhauer ES, Yildiz BO. The prevalence and features of the polycystic ovary syndrome in an unselected population. J Clin Endocrinol Metab. 2004; 89:2745–2749.
3. Goodarzi MO, Quinones MJ, Azziz R, Rotter JI, Hsueh WA, Yang H. Polycystic ovary syndrome in Mexican-Americans: prevalence and association with the severity of insulin resistance. Fertility and sterility. 2005; 84:766–769.
4. Lo JC, Feigenbaum SL, Yang J, Pressman AR, Selby JV, Go AS. Epidemiology and adverse cardiovascular risk profile of diagnosed polycystic ovary syndrome. J Clin Endocrinol Metab. 2006; 91:1357–1363.
5. Okoroh EM, Hooper WC, Atrash HK, Yusuf HR, Boulet SL. Prevalence of polycystic ovary syndrome among the privately insured, United States, 2003-2008. Am J Obstet Gynecol. 2012; 207:299 e291–297.
6. Christensen SB, Black MH, Smith N, Martinez MM, Jacobsen SJ, Porter AH, Koebnick C. Prevalence of polycystic ovary syndrome in adolescents. Fertility and sterility. 2013; 100:470–477.
7. Sirmans SM, Parish RC, Blake S, Wang X. Epidemiology and comorbidities of polycystic ovary syndrome in an indigent population. J Investig Med. 2014; 62:868–874.
8. Moran C, Tena G, Moran S, Ruiz P, Reyna R, Duque X. Prevalence of polycystic ovary syndrome and related disorders in mexican women. Gynecol Obstet Invest. 2010; 69:274–280.
9. Gabrielli L, Aquino EM. Polycystic ovary syndrome in Salvador, Brazil: a prevalence study in primary healthcare. Reprod Biol Endocrinol. 2012; 10:96.
10. Faria FR, Gusmao LS, Faria ER, Goncalves VS, Cecon RS, Franceschini Sdo C, Priore SE. Polycystic ovary syndrome and intervening factors in adolescents from 15 to 18 years old. Rev Assoc Med Bras (1992). 2013; 59:341–346.
11. Michelmore KF, Balen AH, Dunger DB, Vessey MP. Polycystic ovaries and associated clinical and biochemical features in young women. Clin Endocrinol (Oxf). 1999; 51:779–786.
12. Ding T, Baio G, Hardiman PJ, Petersen I, Sammon C. Diagnosis and management of polycystic ovary syndrome in the UK (2004–2014): a retrospective cohort study. BMJ Open. 2016; 6:e012461.
13. Diamanti-Kandarakis E, Kouli CR, Bergiele AT, Filandra FA, Tsianateli TC, Spina GG, Zapanti ED, Bartzis MI. A survey of the polycystic ovary syndrome in the Greek island of Lesbos: hormonal and metabolic profile. J Clin Endocrinol Metab. 1999; 84:4006–4011.
14. Asuncion M, Calvo RM, San Millan JL, Sancho J, Avila S, Escobar-Morreale HF. A prospective study of the prevalence of the polycystic ovary syndrome in unselected Caucasian women from Spain. J Clin Endocrinol Metab. 2000; 85:2434–2438.
15. Sanchon R, Gambineri A, Alpanes M, Martinez-Garcia MA, Pasquali R, Escobar-Morreale HF. Prevalence of functional disorders of androgen excess in unselected premenopausal women: a study in blood donors. Hum Reprod. 2012; 27:1209–1216.
16. Lindholm A, Andersson L, Eliasson M, Bixo M, Sundstrom-Poromaa I. Prevalence of symptoms associated with polycystic ovary syndrome. Int J Gynaecol Obstet. 2008; 102:39–43.
17. Lauritsen MP, Bentzen JG, Pinborg A, Loft A, Forman JL, Thuesen LL, Cohen A, Hougaard DM, Nyboe Andersen A. The prevalence of polycystic ovary syndrome in a normal population according to the Rotterdam criteria versus revised criteria including anti-Mullerian hormone. Hum Reprod. 2014; 29:791–801.
18. Chen X, Yang D, Mo Y, Li L, Chen Y, Huang Y. Prevalence of polycystic ovary syndrome in unselected women from southern China. Eur J Obstet Gynecol Reprod Biol. 2008; 139:59–64.
19. Ma YM, Li R, Qiao J, Zhang XW, Wang SY, Zhang QF, Li L, Tu BB, Zhang X. Characteristics of abnormal menstrual cycle and polycystic ovary syndrome in community and hospital populations. Chin Med J (Engl). 2010; 123:2185–2189.
20. Li R, Zhang Q, Yang D, Li S, Lu S, Wu X, Wei Z, Song X, Wang X, Fu S, Lin J, Zhu Y, Jiang Y, et al. Prevalence of polycystic ovary syndrome in women in China: a large community-based study. Hum Reprod. 2013; 28:2562–2569.
21. Jiao J, Fang Y, Wang T, Wang Z, Zhou M, Wang X. Epidemiologic investigation of polycystic ovarian syndrome (PCOS) in Han ethnic women of reproductive age in Liaoning Province, China. Clin Exp Obstet Gynecol. 2014; 41:304–309.
22. Zhuang J, Liu Y, Xu L, Liu X, Zhou L, Tang L, Kang D, Guo W, He M, Yang F, Qiu D. Prevalence of the polycystic ovary syndrome in female residents of Chengdu, China. Gynecol Obstet Invest. 2014; 77:217–223.
23. Sung YA, Kim DS, Yoo SJ, Baik SY, Oh JY, H.J. L. The prevalence and phenotypes of polycystic ovary syndrome in Korean women. Endocrine Abstracts. 2010; 22:486.
24. Nidhi R, Padmalatha V, Nagarathna R, Amritanshu R. Prevalence of polycystic ovarian syndrome in Indian adolescents. J Pediatr Adolesc Gynecol. 2011; 24:223–227.
25. Gill H, Tiwari P, Dabadghao P. Prevalence of polycystic ovary syndrome in young women from North India: A Community-based study. Indian J Endocrinol Metab. 2012; 16:S389–392.
26. Joshi B, Mukherjee S, Patil A, Purandare A, Chauhan S, Vaidya R. A cross-sectional study of polycystic ovarian syndrome among adolescent and young girls in Mumbai, India. Indian J Endocrinol Metab. 2014; 18:317–324.

27. Kumarapeli V, Seneviratne Rde A, Wijeyaratne CN, Yapa RM, Dodampahala SH. A simple screening approach for assessing community prevalence and phenotype of polycystic ovary syndrome in a semi-urban population in Sri Lanka. Am J Epidemiol. 2008; 168:321–328.

28. Vutyavanich T, Khaniyao V, Wongtra-Ngan S, Sreshthaputra O, Sreshthaputra R, Piromlertamorn W. Clinical, endocrine and ultrasonographic features of polycystic ovary syndrome in Thai women. J Obstet Gynaecol Res. 2007; 33:677–680.

29. Musmar S, Afaneh A, Mo'alla H. Epidemiology of polycystic ovary syndrome: a cross sectional study of university students at An-Najah national university-Palestine. Reprod Biol Endocrinol. 2013; 11:47.

30. Hashemipour M, Faghihimani S, Zolfaghary B, Hovsepian S, Ahmadi F, Haghighi S. Prevalence of polycystic ovary syndrome in girls aged 14–18 years in Isfahan, Iran. Horm Res. 2004; 62:278–282.

31. Mehrabian F, Khani B, Kelishadi R, Ghanbari E. The prevalence of polycystic ovary syndrome in Iranian women based on different diagnostic criteria. Endokrynol Pol. 2011; 62:238–242.

32. Asgharnia M, Mirblook F, Ahmad Soltani M. The Prevalence of Polycystic Ovary Syndrome (PCOS) in High School Students in Rasht in 2009 According to NIH Criteria. Int J Fertil Steril. 2011; 4:156–159.

33. Tehrani FR, Rashidi H, Azizi F. The prevalence of idiopathic hirsutism and polycystic ovary syndrome in the Tehran Lipid and Glucose Study. Reprod Biol Endocrinol. 2011; 9:144.

34. Esmaeilzadeh S, Delavar MA, Amiri M, Khafri S, Pasha NG. Polycystic ovary syndrome in Iranian adolescents. Int J Adolesc Med Health. 2014; 26:559–565.

35. Rashidi H, Ramezani Tehrani F, Bahri Khomami M, Tohidi M, Azizi F. To what extent does the use of the Rotterdam criteria affect the prevalence of polycystic ovary syndrome? A community-based study from the Southwest of Iran. Eur J Obstet Gynecol Reprod Biol. 2014; 174:100–105.

36. Yildiz BO, Bozdag G, Yapici Z, Esinler I, Yarali H. Prevalence, phenotype and cardiometabolic risk of polycystic ovary syndrome under different diagnostic criteria. Hum Reprod. 2012; 27:3067–3073.

37. Khaduri MA, Farsi YA, Najjar TAAA, Gowri V. Hospital-based prevalence of polycystic ovarian syndrome among Omani women. Middle East Fertility Society Journal. 2014; 19:135–138.

38. Attlee A, Nusralla A, Eqbal R, Said H, Hashim M, Obaid RS. Polycystic ovary syndrome in university students: occurrence and associated factors. Int J Fertil Steril. 2014; 8:261–266.

39. Lowe P, Kovacs G, Howlett D. Incidence of polycystic ovaries and polycystic ovary syndrome amongst women in Melbourne, Australia. Aust N Z J Obstet Gynaecol. 2005; 45:17–19.

40. March WA, Moore VM, Willson KJ, Phillips DI, Norman RJ, Davies MJ. The prevalence of polycystic ovary syndrome in a community sample assessed under contrasting diagnostic criteria. Hum Reprod. 2010; 25:544–551.

41. Boyle JA, Cunningham J, O'Dea K, Dunbar T, Norman RJ. Prevalence of polycystic ovary syndrome in a sample of Indigenous women in Darwin, Australia. Med J Aust. 2012; 196:62–66.

42. Joham AE, Ranasinha S, Zoungas S, Moran L, Teede HJ. Gestational diabetes and type 2 diabetes in reproductive-aged women with polycystic ovary syndrome. J Clin Endocrinol Metab. 2014; 99:E447–452.

Supplementary Table 1: Results of prevalence studies (42 studies in total). See Supplementary_ Table_1

Supplementary Table 2: Evaluation of methodological quality of the 42 prevalence studies (score equals the total number of stars). See Supplementary_Table_2

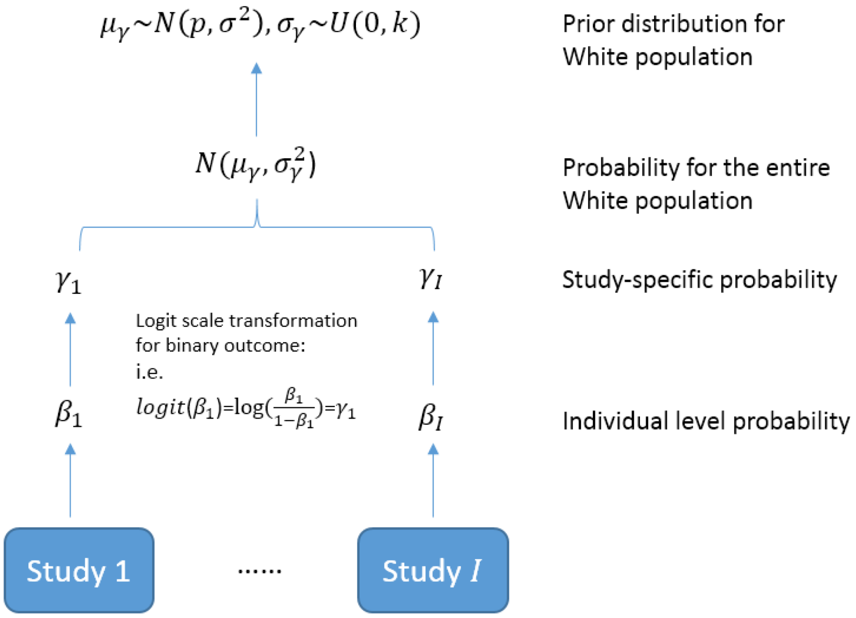

Supplementary Figure 1: The modelling approach is discussed by using an explicit example of the White (Caucasian) population. The results of the rest of ethnic groups were estimated based on the same method. The graphic representation of this model is shown in Figure 1.
