# Supplementary material for: The prevalence of polycystic ovary syndrome in reproductive-aged women of different ethnicity: a systematic review and meta-analysis
Source: Oncotarget. 2017 Jul 12;8(56):96351–8. doi: 10.18632/oncotarget.19180 (PMC5707105; doi:10.18632/oncotarget.19180)
Supplement: Supplementary file 2 [file oncotarget-08-96351-s002.docx]

Supplementary Table 1. Results of prevalence studies (42 studies in total)

| **Article (ref.)** | **Country** | **Sample size** | **Age** | **Estimated Prevalence of PCOS in female population (%)** | | | |
| --- | --- | --- | --- | --- | --- | --- | --- |
|  |  |  |  | **1990 NIH Criteria** | **2003 Rotterdam Consensus** | **2006 Androgen Excess Society** | **Other Criteria** |
| **Americas** |  |  |  |  |  |  |  |
| Knochenhauer *et al*[1]* | US | 277 | 18-45yrs | 4.0 | - | - | - |
| Azziz *et al*  [2]* | US | 400 | 18-45yrs | 6.6 | - | - |  |
| Goodarzi *et al*[3] | US | 156 | 34.0+/- 8.6 yrs | - | - | - | 13 (Self-reported irregular menses and clinical signs of hyperandrogenism) |
| Lo *et al*  [4] | US | 644166 | 15-44yrs | - | - | - | 2.2 |
| Okoroh *et al*[5] | US | 12171830 | 18-45yrs | 1.11 | 1.59 | 1.20 | - |
| Christensen *et al*[6] | US | 137502 | 15-19yrs | 0.56  1.14 (include undiagnosed) | - | - | - |
| Sirman *et al[7]* | US | 143413 | 15-45yrs | - | - | - | 0.88  (ICD-9 code of oligo-menorrhea/amenorrhea plus hirsutism) |
| Moran *et al*[8] | Mexico | 150 | 20-45yrs | 6.0  (95% CI:  1.9-10.1) | 6.6  (95% CI:  2.3-10.9) | - | - |
| Gabrielli *et al*[9]* | Brazil | 859 | 18-45yrs | 8.03 | 8.5 | - | - |
| Faria *et al*  [10] | Brazil | 485 | 15-18yrs | - | - | - | 6.2  (medical diagnosed PCOS) |
| **Europe** |  |  |  |  |  |  |  |
| Michelmore *et al*[11] | UK | 230 | 18-25yrs | 8.0 | - | - | - |
| Ding *et al*[12] | UK | 2,087,107 | 15-45yrs | - | - | - | 2.27 (95% CI 2.23% to 2.31%)  (Readcode defined diagnosis) |
| Diamanti-  Kandarakis *et al*[13]* | Greece | 192 | 17-45yrs | 6.8 | - | - | - |
| Asuncion *et al*[14]* | Spain | 154 | 18-45yrs | 6.5 | - | - | - |
| Sanchón *et al*  [15]* | Spain | 592 | ≥ 18yrs  Median:  27~33*  IQR:  9~13* | 5.4%  (95% CI:  3.6-7.2) | - | - | - |
| Lindholm *et al*  [16] | Sweden | 147 | 25-40yrs | - | - | - | 4.8  (self-reported 1990 NIH) |
| [Lauritsen](http://www.ncbi.nlm.nih.gov/pubmed?term=Lauritsen%20MP%5BAuthor%5D&cauthor=true&cauthor_uid=24435776) *et al*[17] | Denmark | 447 | 20-40yrs | - | 16.6 | - | - |
| **Asia** |  |  |  |  |  |  |  |
| Chen *et al*  [18]* | China | 915 | 19-45yrs | - | 2.4 | 2.2 | - |
| Ma *et al*  [19]* | China | 2111 | 19-45yrs | - | 6.11 | - | - |
| [Li](http://www.ncbi.nlm.nih.gov/pubmed?term=Li%20R%5BAuthor%5D&cauthor=true&cauthor_uid=23814096) *et al*  [20]* | China | 15924 | 19-45yrs | - | 5.6 | - | - |
| [Jiao](http://www.ncbi.nlm.nih.gov/pubmed?term=Jiao%20J%5BAuthor%5D&cauthor=true&cauthor_uid=24992782)*et al*  [21]* | China | 1600 | 19-45yrs | - | 8.25 | - | - |
| [Zhuang](http://www.ncbi.nlm.nih.gov/pubmed?term=Zhuang%20J%5BAuthor%5D&cauthor=true&cauthor_uid=24751759)*et al*  [22] | China | 1645 | 12-44yrs | 7.1 | 11.2 | 7.4 | - |
| Sung *et al*  [23] | Korea | 8080 (target) | 16-39yrs | 4.4 | 6.3 | 5.1 | - |
| Nidhi *et al*  [24] | India | 460 | 15-18yrs | - | 9.13  10.97 (imputation) | - | - |
| Gill *et al*  [25] | India | 1520 | 18-25yrs | 3.7 | - | - | - |
| [Joshi](http://www.ncbi.nlm.nih.gov/pubmed?term=Joshi%20B%5BAuthor%5D&cauthor=true&cauthor_uid=24944925) *et al*  [26] | India | 600 | 15-24yrs | - | 22.5 | 10.7 | - |
| Kumarapeli *et al*[27] | Sri Lanka | 2915 | 15-39yrs | - | 6.3  (95% CI:  5.9-6.8) | - | - |
| Vutyavanich et al [28] | Thailand | 1095 | 18-40yrs | 5.7 | - | - | - |
| **Middle East** |  |  |  |  |  |  |  |
| Musmar *et al*[29] | Palestine | 137 | 18-24yrs | 7.3 | - | - | - |
| [Hashemipour](http://www.ncbi.nlm.nih.gov/pubmed?term=Hashemipour%20M%5BAuthor%5D&cauthor=true&cauthor_uid=15523185) *et al*[30] | Iran | 1000 | 14-18yrs | - | - | - | 3 (clinical PCOS) |
| Mehrabian *et al*[31] | Iran | 820 | 17-34yrs | 7.0 | 15.2 | 7.92 | - |
| [Asgharnia](http://www.ncbi.nlm.nih.gov/pubmed?term=Asgharnia%20M%5BAuthor%5D&cauthor=true&cauthor_uid=24851175) *et al*[32] | Iran | 1850 | 17-18yrs | 11.34 | - | - | - |
| [Tehrani](http://www.ncbi.nlm.nih.gov/pubmed?term=Tehrani%20FR%5BAuthor%5D&cauthor=true&cauthor_uid=21435276) *et al*[33]* | Iran | 929 | 18-45yrs | 7.1  (95% CI:  5.4-8.8) | 14.6  (95% CI:  12.3-16.9) | 11.7  (95% CI:  9.5-13.7) | - |
| Esmaeilzadeh *et al*[34] | Iran | 1549 | 16-20yrs | - | - | - | 8.3 (95% CI: 4.0-12.0, criteria of PCOS not stated) |
| Rashidi *et al*[35]* | Iran | 602 | 18-45yrs | 4.8  (95% CI:  3.1-6.5) | 14.1  (95% CI:  11.3-16.9) | 12.0  (95% CI:  9.3-14.5) | - |
| Yildiz *et al[36]** | Turkey | 392 | 18-45yrs | 6.1 | 19.9 | 15.3 | - |
| Khaduri *et al*  [37] | Oman | 3644 | 12-45yrs | - | 7.0 | - | 2.8 (95% CI: 0.7-9.6, per 1000 in 2010). |
| Attlee *et al*  [38] | United Arab Emirates | 50 | 17-23yrs | - | - | - | 20 (criteria of PCOS not stated) |
| **Oceania** |  |  |  |  |  |  |  |
| Lowe *et al*  [39] | Australia | 100 | - | - | 12 | - | - |
| March *et al*[40] | Australia | 728 | 27-34yrs | 8.7+/-2.0  (95% CI) | 11.9 +/- 2.4  17.8 +/- 2.8 (imputation for non-consenting group included) | 10.2 +/- 2.2  12.0+/- 2.4 (imputation for non-consenting group included) | - |
| [Boyle](http://www.ncbi.nlm.nih.gov/pubmed?term=Boyle%20JA%5BAuthor%5D&cauthor=true&cauthor_uid=22256938)*et al*[41] | Australia | 248 | 15-44yrs | 15.3  (95% CI,  10.8–19.8) | - | - | - |
| Joham *et al*  [42] | Australia | 8612 | 28-33yrs | - | - | - | 5.8 (95% CI: 5.3-6.3, self-reported PCOS). |
