# Supplementary material for: The prevalence of polycystic ovary syndrome in reproductive-aged women of different ethnicity: a systematic review and meta-analysis
Source: Oncotarget. 2017 Jul 12;8(56):96351–8. doi: 10.18632/oncotarget.19180 (PMC5707105; doi:10.18632/oncotarget.19180)
Supplement: Supplementary file 3 [file oncotarget-08-96351-s003.docx]

Supplementary Table 2: Evaluation of methodological quality of the 42 prevalence studies (score equals the total number of stars)

| Study | Appropriate sampling † | PCOS measured reliably and objectively ‡ | Response rate ◊ | Sample size ∆ | Crude number  of cases $ | Age range§ | Ethnicity Ƨ | Score |
| --- | --- | --- | --- | --- | --- | --- | --- | --- |
| Knochenhauer et al (1998)[1] | × | * | * | * | * | * | * | 6 |
| Michelmore et al (1999)[11] | × | * | × | * | × | × | * | 3 |
| Diamanti-Kandarakis et al (1999)[13] | × | * | * | * | * | * | * | 6 |
| Asuncion et al (2000)[14] | × | * | * | * | * | * | * | 6 |
| Azziz et al(2004)[2] | × | * | × | * | * | * | * | 5 |
| [Hashemipour](http://www.ncbi.nlm.nih.gov/pubmed?term=Hashemipour%20M%5BAuthor%5D&cauthor=true&cauthor_uid=15523185) et al (2004)[30] | * | * | * | * | * | × | * | 6 |
| Goodarzi et al (2005)[3] | × | × | * | * | * | * | * | 5 |
| Lowe et al (2005)[39] | × | × | * | * | * | × | × | 3 |
| Lo et al (2006)[4] | × | × | × | × | × | * | × | 1 |
| Vutyavanich et al (2007)[28] | × | * | * | * | * | * | * | 6 |
| Chen et al (2008)[18] | × | * | * | * | * | * | * | 6 |
| Kumarapeli et al (2008)[27] | * | * | * | * | * | * | * | 7 |
| Lindholm et al (2008)[16] | * | * | * | * | * | × | * | 6 |
| Yildiz et al (2012)[36] | × | * | * | * | * | * | * | 6 |
| March et al (2010)[40] | × | * | × | * | * | × | * | 4 |
| Ma et al (2010)[19] | * | * | * | * | * | * | * | 7 |
| [Moran](http://www.ncbi.nlm.nih.gov/pubmed?term=Moran%20C%5BAuthor%5D&cauthor=true&cauthor_uid=20110726) et al (2010)[8] | × | * | * | * | * | * | * | 6 |
| Sung et al (2010)[23] | × | * | × | × | × | * | × | 2 |
| [Asgharnia](http://www.ncbi.nlm.nih.gov/pubmed?term=Asgharnia%20M%5BAuthor%5D&cauthor=true&cauthor_uid=24851175) et al (2011)[32] | * | * | * | * | * | × | * | 6 |
| Mehrabian et al (2011)[31] | × | * | × | * | * | × | * | 4 |
| Nidhi et al (2011)[24] | × | * | * | * | * | × | * | 5 |
| [Tehrani](http://www.ncbi.nlm.nih.gov/pubmed?term=Tehrani%20FR%5BAuthor%5D&cauthor=true&cauthor_uid=21435276) et al (2011)[33] | * | * | * | * | * | * | * | 7 |
| [Boyle](http://www.ncbi.nlm.nih.gov/pubmed?term=Boyle%20JA%5BAuthor%5D&cauthor=true&cauthor_uid=22256938) et al (2012)[41] | × | * | * | * | * | * | * | 6 |
| [Gabrielli](http://www.ncbi.nlm.nih.gov/pubmed?term=Gabrielli%20L%5BAuthor%5D&cauthor=true&cauthor_uid=23173761) et al (2012)[9] | * | * | * | * | * | * | * | 7 |
| Gill et al (2012)[25] | × | * | × | * | * | × | * | 4 |
| Okorohet al (2012) [5] | × | × | × | * | × | * | × | 2 |
| Sanchón et al (2012)[15] | × | ***** | ***** | ***** | ***** | ***** | × | 5 |
| [Christensen](http://www.ncbi.nlm.nih.gov/pubmed?term=Christensen%20SB%5BAuthor%5D&cauthor=true&cauthor_uid=23756098) et al (2013)[6] | × | × | × | * | * | × | × | 2 |
| Esmaeilzadeh et al (2014)[34] | * | * | * | * | * | × | * | 6 |
| Faria et al (2013)[10] | × | × | × | * | * | × | × | 2 |
| Joham et al (2014)[42] | * | × | × | * | * | × | * | 4 |
| Khaduri et al (2013)[37] | × | × | × | * | * | * | × | 3 |
| [Li](http://www.ncbi.nlm.nih.gov/pubmed?term=Li%20R%5BAuthor%5D&cauthor=true&cauthor_uid=23814096) et al (2013)[20] | * | * | * | * | * | * | * | 7 |
| Musmar et al (2013)[29] | × | * | * | * | * | × | * | 5 |
| Attlee et al (2014)[38] | × | × | * | * | * | × | * | 4 |
| Lauritsen et al (2014)[17] | × | * | × | * | * | * | * | 5 |
| [Jiao](http://www.ncbi.nlm.nih.gov/pubmed?term=Jiao%20J%5BAuthor%5D&cauthor=true&cauthor_uid=24992782) et al (2014)[21] | × | * | × | * | * | * | * | 5 |
| [Joshi](http://www.ncbi.nlm.nih.gov/pubmed?term=Joshi%20B%5BAuthor%5D&cauthor=true&cauthor_uid=24944925) et al (2014)[26] | * | * | * | * | * | × | * | 6 |
| [Rashidi](http://www.ncbi.nlm.nih.gov/pubmed?term=Ramezani%20Tehrani%20F%5BAuthor%5D&cauthor=true&cauthor_uid=25224635)et al (2014)[35] | * | * | * | * | * | * | * | 7 |
| Sirman et al (2014)[7] | × | × | × | * | * | * | * | 4 |
| [Zhuang](http://www.ncbi.nlm.nih.gov/pubmed?term=Zhuang%20J%5BAuthor%5D&cauthor=true&cauthor_uid=24751759) et al (2014)[22] | * | * | * | * | * | × | * | 6 |
| Ding et al (2016)[12] | × | × | × | * | * | * | × | 3 |

† (a) Appropriate sampling (target population clearly defined and probability sampling applied) (awarded one star)

(b) Inappropriate sampling (i.e. convenient sampling) **OR** cases based on medical records where sampling frame was not applied **OR** not stated/unknown (cross)

‡ (a) Systematic screening performed for sample population and PCOS was strictly defined (awarded one star)

(b) Medical records based studies where no systematic screening was performed **OR** studies which used self-reported (i.e. based on questionnaire) PCOS cases

◊(a)Low non-participant rate (≤30%) of the initial target sample population for further study (i.e. systematic screening) **OR** low non-response rate (≤30%) of a deliberated-designed questionnaire (awarded one star)

(b) High non-response rate or refusal rate to further study (≥30%) of the initial target sample population **OR** high non-response rate (≥30%) of a deliberated-designed questionnaire OR studies based on electronic medical record where incomplete patient information (i.e. missing data) is a routine problem, leading to incomplete ascertainment of cases **OR** not stated/unknown (cross)

∆ (a) Sample size clearly stated (awarded one star) (b) Not stated/unknown (cross)

$ (a) Crude number of cases clearly stated (awarded one star) (b) Not stated/unknown (cross)

§ (a) Age range of the sample population is approximately same as the reproductive age, i.e. 15~45 years, 18~45 years, 17~45 years (awarded one star)

(b) Otherwise (narrower age range **OR** upper/lower bound of age range lying outside the limit, i.e. 18~24 years, 12~44 years **OR** not stated/unknown) (cross)

Ƨ (a) Ethnicity of PCOS cases and sample population clearly stated (awarded one star)

(b) Ethnicity not clearly stated/unknown (cross)
